# Effectiveness of Ankle Proprioceptive Neuromuscular Facilitation Techniques in Restoring the Biomechanical Integrity of the Ankle Following Plantar Fasciitis: An Experimental Study

**DOI:** 10.7759/cureus.64371

**Published:** 2024-07-11

**Authors:** Anam R Sasun, Tejal Babar, Ragini Dadgal

**Affiliations:** 1 Department of Neuro-Physiotherapy, Ravi Nair Physiotherapy College, Datta Meghe Institute of Higher Education and Research (DMIHER), Wardha, IND

**Keywords:** plantar fasciitis, neurophysiotherapy rehabilitation, pnf, experimental study, balance

## Abstract

Background

Recent studies have highlighted the role of the central nervous system in modulating pain perception and the movement patterns associated with plantar fasciitis. Neurological changes, such as altered sensorimotor control and cortical reorganization, may contribute to the persistence of symptoms and the recurrence of the condition. Integrating neurorehabilitation techniques may enhance outcomes and reduce the risk of recurrence. Physiotherapy exercises such as ankle proprioceptive neuromuscular facilitation, foot doming exercises, balance exercises, towel curl exercises, and stretching exercises were given to check the impact of physiotherapy interventions on ankle muscle instability and dynamic balance following plantar fasciitis.

Method

An experimental investigation was carried out at the outpatient department of Acharya Vinoba Bhave Rural Hospital. A total of 71 participants were assigned arbitrarily, employing a straightforward random sampling procedure. Each participant received treatment for six weeks, with five weekly sessions.

Result

The results demonstrated significant findings. The pre- and post-test score results are as follows: visual analogue scale scores (t=1.619, p=0.0001), weight-bearing lunge test scores (t=24.36, p=0.0001*), and functional reach test scores (t=24.36, p=0.0001).

Conclusion

We conclude that physiotherapy exercises such as ankle proprioceptive neuromuscular facilitation (PNF), foot doming exercises, strengthening exercises, toe spreading exercises, towel curl exercises, and stretching exercises are effective in reducing pain and ascertaining dynamic balance in plantar fasciitis. The rehabilitation program significantly improved ankle biomechanical integrity and muscle strength, allowed functional recovery, and reduced pain. Future studies should focus on investigating the long-term effects of PNF therapies. For better patient outcomes, clinicians should consider incorporating ankle PNF exercises into their therapy regimens.

## Introduction

Plantar fasciitis is marked by inflammation in the tissues surrounding the calcaneus tuberosity, known as the plantar fascia. Improper footwear selections are a major contributing factor in the emergence of foot discomfort, which has a complex etiology. Foot pain has been related to wearing shoes that lack support and stability [1]. Repetitive irritation leads to joint laxity, subsequently causing mechanical instability in the ankle joint and resulting in an ankle injury [2]. Plantar fasciitis is commonly thought to be caused by mechanical overload and excessive tension on the plantar fascia. This alteration in ankle stability affects the musculature, complicating matters by influencing dynamic balance [3]. Plantar fasciitis can be brought on by mechanical overload and inappropriate footwear. Plantar fasciitis can cause pain and change biomechanics that can modify gait patterns and cause compensatory motions that impair balance and raise the risk of ankle injury.

Currently, there is a scarcity of literature assessing the impact of physiotherapy interventions on ankle muscle instability and dynamic balance following plantar fasciitis. The distribution of forces and loads during walking is altered by plantar fasciitis, which impacts the ankle joint's biomechanics. Pain and inflammation can cause compensatory alterations in gait mechanics, leading to aberrant ankle movements and increased joint stress. These compensatory responses can lead to decreased mobility and changed alignment, which exacerbates ankle instability and impairs dynamic balance. The plantar fascia experiences increasing loading stress that flattens the longitudinal arch and induces foot eversion if the musculatures supporting it are weak and the fascia is not flexible. As a result, the impacted side hip falls, causing an inward rotation of the hip and narrowing the space between the knee joints [4]. This sequence may overload the plantar fascia, create muscle imbalances, and disrupt normal gait, ultimately impacting the dynamic balance of an individual and resulting in diminished functional performance.

Proprioceptive neuromuscular facilitation (PNF) is widely applied in the fields of exercise and sports to enhance functions. In particular, ankle PNF focuses more on time to nurture and synchronize the foot's internal and external muscle action patterns [5]. According to a systematic review and meta-analysis conducted in 2022, engaging in foot and ankle muscle strengthening exercises can positively impact joint position sense in individuals with chronic ankle instability (CAI) [6]. However, it is worth noting that as the balance exercise intervention becomes more intricate, the effectiveness of proprioceptive outcomes tends to diminish [7].

A scarcity of literature exists for assessing the impact of physiotherapy interventions on ankle muscle instability and dynamic balance after plantar fasciitis when scrutinizing the available research. The substantial influence of ankle muscle instability on balance, gait, synergy, and posture is often disregarded in many rehabilitation approaches. This highlights the opportunity for devising a specialized protocol that systematically addresses the imperative to restore strength in ankle joint muscles. The research explores whether physiotherapy therapies can effectively restore muscular strength and dynamic stability post-plantar fasciitis. The study aims to identify the effect of ankle proprioceptive neuromuscular facilitation techniques on the biomechanics of the ankle joint and the vitality biomechanics of the ankle joint in optimizing the static and dynamic balance in patients with plantar fasciitis.

## Materials and methods

Study design and setting

The study was conducted after receiving approval from the Institutional Ethics Committee (IEC) of Datta Meghe Institute of Medical Sciences (DMIMS), Deemed to be a University (DU) (ethical clearance number DMIMS(DU)/IEC/2021/382). The study's research design is an interventional study, whereas the study design is an experimental study. The study was conducted in two healthcare facilities in Wardha, India: the neuro-physiotherapy department of Ravi Nair Physiotherapy College and the neuro-physiotherapy outpatient department of Acharya Vinoba Bhave Rural Hospital. All patients received treatment at these locations.

Participant selection

A total of 71 participants were included in the study. A simple random sampling technique for randomization processes was used. A computer-generated list served as the basis for the allocation process. The participants were selected based on the inclusion and exclusion criteria shown in Table 1.

**Table 1:** The inclusion and exclusion criteria of the study

Inclusion Criteria	
1.	Participants were diagnosed with plantar fasciitis
2.	Exacerbating pain at the calcaneal tubercle and the heel of the foot in the morning, lasting three months and longer
3.	Positive for Windlass test
4.	Age 20–40 years
Exclusion Criteria	
1.	Undiagnosed pain in the foot, those undergoing steroid treatment for plantar fasciitis
2.	Numbness and tingling in the lower extremities with or without provocation
3.	Pregnant women

Treatment procedure

The study comprised individuals who met the eligibility requirements and indicated their desire to participate after screening them based on the inclusion and exclusion criteria. The individuals were treated for six weeks, with five weekly sessions. A qualified physiotherapist conducted the session. Each session lasted for 60 minutes. Ankle rhythmic initiation was employed for the ankle proprioceptive neuromuscular facilitation technique. The physiotherapy included exercises presented in Table 2.

**Table 2:** The description of the physiotherapy rehabilitation for the patient ex: exercises; PNF: proprioceptive neuromuscular facilitation; TA: tendoachilles; sec: seconds

Foot Exercises	1-2 Weeks	3-4 Weeks
Heel raise ex	2×10 times	4×10 times
Heel raise ex	2×10 times	5×10 times
Towel curls	4×10 times	5×10 times
Foot doming ex	2×10 times	5×10 times
Toe spread ex	3×10 times	5×10 times
Wobble board ex	2×10 times	5×10 times
Balance ex	3×10 times	6×10 times
Stretching for TA, investors, inventors, plantar-dorsiflexors	5×10 times-15 sec hold	5×10 times-20 sec hold
Ankle PNF (rhythmic-stabilization)	3-reps, 90-sec duration	4–reps, 90-sec duration
Resisted-eccentric inversion ex	3×10 times	5×10 times
Proprioception ankle ex	4×10 times	5×10 times

Outcome measures

The weight-bearing lunge test (WBLT) [8] is a useful tool for understanding how the lower extremities operate, particularly for those who have plantar fasciitis. This entails modifying the exam to account for any pain or limited movement related to the ailment, guaranteeing a secure and efficient assessment procedure. The visual analogue scale (VAS) [9] is a commonly used tool for measuring pain intensity. This scale often consists of a line, horizontal or vertical, with ends that represent the range of pain levels (from "no pain" to "worst pain imaginable"). The functional reach test (FRT) [10] is a measure of an individual's capacity to reach forward without stepping and to maintain balance while assessing dynamic balance and stability. Despite not being expressly created for the assessment of plantar fasciitis, it offers important insights into the function and balance of the lower extremities. These aspects can be affected by the ailment.

Statistical analysis

The statistical analysis encompassed both descriptive and inferential statistics, employing Student's paired and unpaired t-tests. The software applications employed for this analysis included GraphPad Prism, Version 7.0 (GraphPad Software, San Diego, California) and IBM SPSS Statistics for Windows, Version 27 (Released 2020; IBM Corp., Armonk, New York). The predetermined significance level was established at a p-value of less than 0.05. In examining age-wise distribution, an unpaired t-test was employed to compare the post-mean between groups. Simultaneously, a paired t-test was utilized to assess the variance between pre- and post-data within the specified group.

## Results

The level of significance is considered at p<0.05. Of the total patient population, 28.17% were in the age groups of 20-25 years and 36-40 years, 28.17% were in the age group of 20-25 years, 23.94% were in the age group of 26-30 years, 19.72% of the patients were in the age group of 31-35 years, and 28.17% were in the age group 36-40 years. The mean age of the patients was 30.52 ± 6.30. Table 3 presents the age distribution of the patients.

**Table 3:** Age distribution of the patients (years)

Age Group (Years)	Number of Patients	Percentage
20-25 years	20	28.17
26-30 years	17	23.94
31-35 years	14	19.72
36-40 years	20	28.17
Total	71	100

Functional reach test

Before treatment, the mean FRT score was 5.32±1.46; after treatment, it was 9.38±1.75. A significant variation in the FRT scores of the patients was discovered using the Student's paired t-test (t=24.36, p=0.0001, significant) (Table 4).

**Table 4:** Mean pre- and post-test scores of the functional reach test *p=0.0001 implies statistically significant results

	Mean	Participants (n)	Standard Deviation	Standard Error Mean	Mean Difference
Pre-test	5.32	71	1.46	0.17	1.05±1.40
Post-test	9.38	71	1.75	0.20	
t-value	24.36 (p=0.0001*), significant				

Visual analogue scale

Modifications in VAS scores indicate changes in the severity of a patient's pain, which makes them clinically significant. Significantly lower VAS scores are associated with better patient outcomes, such as better pain control and a higher standard of living. Pain reduction can result in improved mobility, functional performance, and general well-being; therefore, VAS score modifications are an important indicator of treatment effectiveness. The mean VAS score was 7.77±1.22 before treatment, and it was 4.15±1.29 following treatment. The Student's paired t-test found a statistically significant variance (t=1.619, p-0.0001) between the VAS scores (Table 5).

**Table 5:** Visual Analogue Scale scores *p=0.0001 implies statistically significant values

	Mean	Participants (N)	Standard Deviation	Standard Error Mean	Mean Difference
Pre-test	7.77	71	1.22	0.145	0.191
Post-test	4.15	71	1.29	0.191	
t-value	1.619 (p=0.0001*), significant				

Weight-bearing lunge test

**Figure 1:**
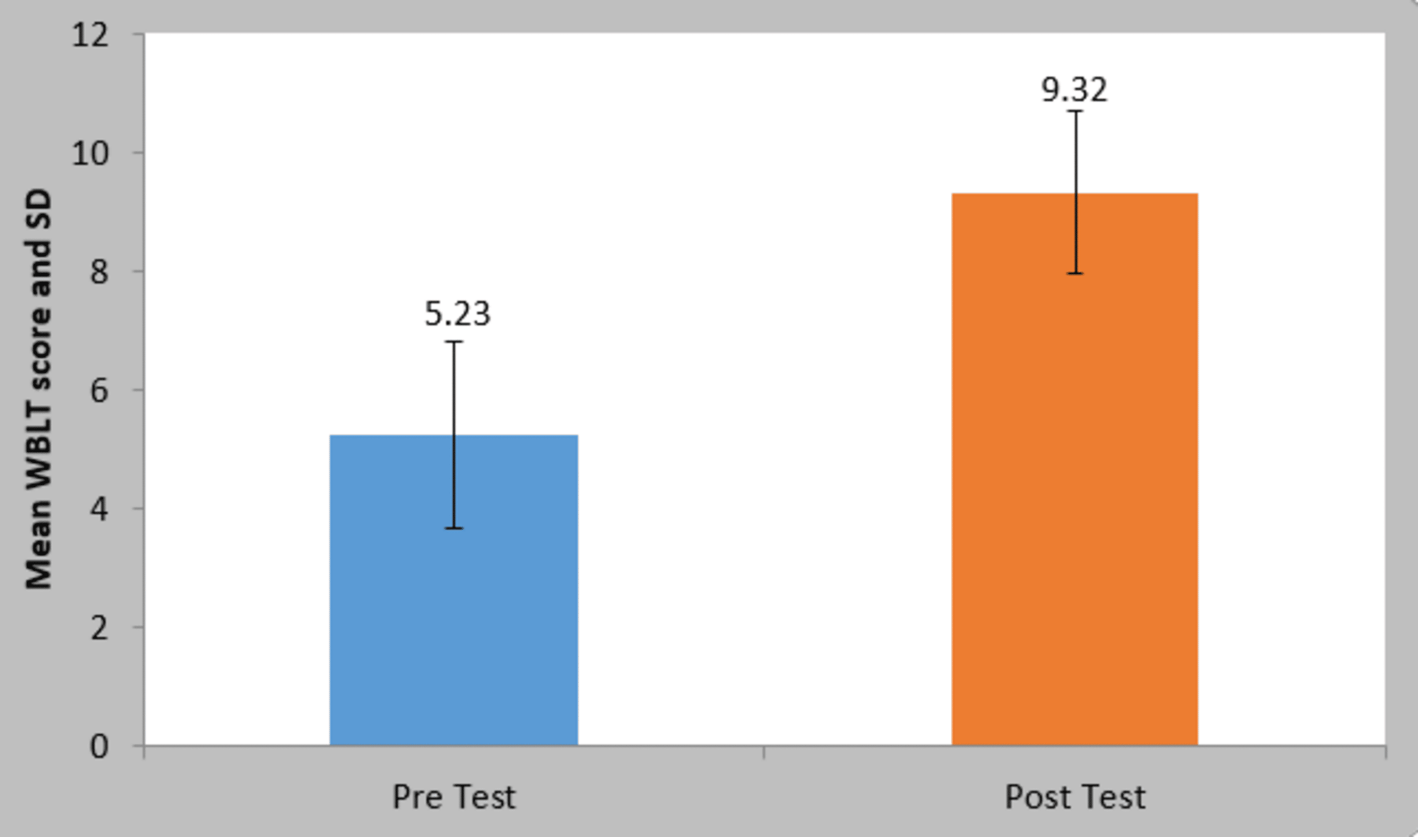
Comparison of weight-bearing lunge test scores p=0.0001 implies statistically significant results SD: standard deviation; WBLT: weight-bearing lunge test

Before treatment, the mean WBLT score was 5.23±1.46; after treatment, it was 9.32±1.75. A statistically significant difference between the WBLT scores of the patients before and after therapy was observed (t=24.36, p=0.0001) (Figure 1).

## Discussion

The study aims to evaluate the effect of physiotherapy interventions on ankle muscle instability and dynamic balance following plantar fasciitis. Those with plantar fasciitis who have aberrant foot posture, such as a flat foot, might encounter prolonged plantar fascia stretching due to losing their foot arch, further damaging the plantar fascia [11]. A modified star excursion is used to check the balance [12]. This is known to cause excessive strain on the muscles and joints of the lower extremities [13]. According to Rozzi et al. (1999), implementing a rehabilitation strategy to address the repercussions of the injury on the articulation of the ankle joint holds the potential for mitigating the development of CAI. Various exercises, including deep squats, wobble board exercises, resistance band kicks, in-line lunges, single-leg foam balance exercises, coordination training, and the Hurdle Step, have demonstrated efficacy in enhancing balance and strengthening among patients [14]. Ranbhor et al. (2021) concluded that foam rollers gave statistically significant results in decreasing the pain threshold of the soleus and gastrocnemius.

Moreover, Hyland et al. (2006) published an RCT (randomized controlled trial) stating the positive effects of calcaneal taping over stretching on reducing plantar heel pain in plantar fasciitis [15]. Further, Shashua et al. (2015) concluded a study stating the positive impact of mobilization on decreasing pain and increasing the ankle's range of motion. Additionally, Lee et al. (2020) noted the positive effects of combined isotonic training on improving pain, muscular strength, and foot function in obese individuals with plantar fasciitis [4]. According to this meta-analysis, low-level laser therapy significantly decreases heel pain in individuals with plantar fasciitis, and its efficacy lasts for three months following laser treatment [16]. During their investigation, subjects experiencing CAI observed that dynamic balance, compared to static balance, presented a heightened challenge to the central nervous system. The researchers recommended evaluating differences through a single measurement to ensure reliable assessments. When CAI acts as an obstacle for the central nervous system, communication within the neural network is impeded, resulting in a limitation in the formation of essential patterns to address alterations in direction or sudden shifts in inertia [17]. Compared to uninjured contralateral limbs and healthy individuals, the injured ankles of patients with CAI showed impairment in proprioception (kinaesthesia and joint position sense). The degree to which proprioceptive abnormalities were evident varied depending on specific movement directions and testing methods. When treating CAI clinically, more complex proprioceptive assessments and therapies targeted at regaining these deficits should be used [18].

Songlin et al. (2023) conducted a narrative review study to study the repercussions of tDCS (transcranial-direct current stimulation). The results revealed that tDCS can potentially enhance foot biomechanics in healthy adults. Moreover, in foot sports medicine, tDCS emerges as a promising adjunctive tool, particularly when given functional training. It improves foot biomechanical performance. This improvement is believed to stem from the ability of tDCS to stimulate specific neurons pertinent to the task at hand and modulate various neurons within the neural system, thereby influencing foot biomechanical characteristics positively [19]. Shashua et al. (2015) published a randomized controlled study to study the effects of ankle and mid-foot mobilization on plantar fasciitis [20]. The study concluded by stating the positive impact of ankle and mid-foot mobilization on plantar fasciitis. Tezel et al. concluded Kinesio taping is more effective than extracorporeal therapy in reducing pain and symptoms in plantar fasciitis [21]. Further studies should include interventions to enhance the ability of the nervous system to modulate biomechanical functions. Also, subsequent investigations should employ multimodal neuroimaging techniques to delve into the intrinsic ergogenic mechanism underlying the effects of tDCS.

The limitations of the study are the small sample size of 71 participants and the lack of follow-up for the patients. Further, this study did not investigate the ergogenic mechanism of tDCS. Future studies should consider these factors for further research.

## Conclusions

Plantar fasciitis can disturb balance dynamics, potentially heightening the risk of falls. The condition's influence on the plantar fascia's elasticity may contribute to balance impairments, while delays in tension development across the foot can provoke motor errors and exacerbate tremors, diminishing stability further. It is imperative to acknowledge these intricacies when addressing balance concerns in individuals with plantar fasciitis and to provide tailored information and guidance to mitigate fall risk. Therefore, this experimental study was conducted to examine the effectiveness of these physiotherapy interventions. Our analysis indicates that physiotherapy therapies had a substantial positive impact on reducing pain, increasing strength, and improving balance in patients diagnosed with plantar fasciitis. It also showed improvement in the outcome measures used, particularly the WBLT, VAS, and FRT.
